# Low cutaneous follistatin expression is associated with striae gravidarum: A pilot study of the TGF-β/BMP signaling pathway

**DOI:** 10.1016/j.bbrep.2026.102842

**Published:** 2026-10-13

**Authors:** Tian Tian, Huiyu Wang, Yuanhong Li

**Affiliations:** a Department of Dermatology, General Hospital of Ningxia Medical University, Yinchuan, Ningxia, 750004, China; b Ningxia Joie Material Co., Ltd, Yinchuan, Ningxia, 751400, China; c Department of Dermatology, China Medical University, Shenyang, Liaoning, 110122, China

**Keywords:** Follistatin, Molecular pathogenesis, TGF-β/BMP signaling, Striae gravidarum

## Abstract

Striae gravidarum (SG) causes prominent cosmetic impairment and psychological distress, yet its underlying etiology and molecular mechanisms remain poorly characterized. Unraveling core molecular cascades of SG is critical for developing targeted therapeutics. In this study, we first screened differentially expressed genes among the TGF-β/BMP-related molecules in skin biopsies from four SG patients and six unaffected postpartum controls via TGF-β/BMP-focused PCR Array to prioritize candidate pathogenic genes. *F*ollistatin (*FST*) emerged as the most prominently downregulated candidate gene in SG, with expression reduced by approximately 225.59-fold in SG lesional skin versus non-SG control skin. We further validated the expression profiles of FST and core related molecules (TGF-β1, BMP4 and Smad3) using molecular assays. Quantitative analyses revealed consistent downregulation of all these molecules in lesional SG skin. Immunohistochemical staining was additionally applied to verify the tissue-level expression pattern of FST. Taken together, our results suggest that low cutaneous FST expression may be associated with the pathogenesis of striae gravidarum.

## Highlights

• FST shows a prominent expression difference in postpartum striae gravidarum.
• TGF-β/BMP-Smad3-related molecular abundance changes are validated by PCR and western blot assays.
• IHC reveals altered dermal FST expression in striae gravidarum skin samples.
• This pilot study provides preliminary clues related to striae gravidarum pathogenesis.

## 1 Introduction

Striae gravidarum (SG) frequently develops in late pregnancy, affecting approximately 56% of pregnant women, with typical dermal collagen and elastic fiber damage [1]. Despite lacking physical symptoms, SG compromises appearance and mental well-being, reducing patients’ life quality [2]. To date, the exact etiology and molecular pathogenesis of SG remain incompletely defined, mechanistic investigations into stria formation at the molecular level remain scarce. Therefore, elucidating the molecular pathogenesis of SG is essential and may provide valuable theoretical evidence for improving clinical prevention and therapeutic strategies.

Follistatin (FST) is a widely expressed secreted glycoprotein that primarily binds activin and modulates multiple downstream signaling cascades of the TGF-β superfamily, including BMP-mediated pathways [3]. Accumulating evidence from prior studies suggests that FST participates in the endogenous regulation of the TGF-β/BMP signaling axis, with important roles in governing dermal extracellular matrix (ECM) homeostasis and skin remodeling. Consistent with this notion, previous animal studies have demonstrated that *FST* deficiency leads to impaired cutaneous structure and embryonic lethality, strongly supporting an indispensable role of FST in maintaining skin tissue stability [4]. The TGF-β signaling pathway is well established as a central regulator of dermal ECM remodeling [5], and its dysregulation has been implicated in fibrotic and atrophic skin disorders characterized by typical striae-like lesions, such as Marfan syndrome [6].

We hypothesized that altered expression of TGF-β/BMP-related molecules may correlate with SG development, and that FST may be associated with these molecular changes. To test this hypothesis, we adopted a TGF-β/BMP pathway-focused PCR Array to compare gene expression profiles in skin tissues from SG patients and matched postpartum controls. Afterwards, we validated candidate pathway molecules at mRNA, protein and in situ tissue levels via qPCR, Western blot and immunohistochemistry. This study aimed to characterize the expression pattern of FST and TGF-β/BMP-related molecules in SG skin, so as to provide novel molecular clues for exploring the mechanisms underlying SG.

## 2 Materials and methods

### 2.1 Samples

Skin specimens were obtained from four postpartum patients with abdominal striae gravidarum and six age- and weight-matched postpartum healthy controls, all recruited from the dermatology outpatient clinic. This study was approved by the local Ethics Committee and conducted in accordance with the Declaration of Helsinki. Written informed consent was obtained from all participants.

### 2.2 PCR array analysis

Total RNA was extracted from skin tissues using the miRNeasy Mini Kit (Qiagen). RNA purity and concentration were determined by NanoDrop spectrophotometry. TGF-β/BMP pathway gene screening was performed using a human pathway-focused PCR Array (Qiagen) following the manufacturer's protocols. Fold change was calculated as the ratio of gene expression level in SG lesional skin to that in non-SG control skin. For downregulated genes, the fold reduction value was calculated as the reciprocal of the SG/control ratio.

### 2.3 Quantitative Real-Time PCR

For validation of candidate genes, quantitative Real-Time PCR (qPCR) was performed to detect FST, TGF-β1, BMP4 and Smad3 mRNA expression. Reverse transcription and amplification were conducted using commercial kits (Promega). Gene-specific primer sequences are shown below: *FST* (forward: 5′-GACCGAGGAGGACGTGAATG-3′, reverse: 5′-ACAGTCCACGTTCTCACACG-3′), *TGF-β1* (forward: 5′-TCCTGGCGATACCTCAGCAA-3′, reverse: 5′-CCGGTAGTGAACCCGTTGAT-3′), *BMP4* (forward: 5′-GAGGAGGAGGAGGAAGAGCA-3′, reverse: 5′-TGCTGCTGAGGTTGAAGAGG-3′), *SMAD3* (forward: 5′-CATGGACGCAGGTTCTCCAA-3′, reverse: 5′-TCCACTGCTGCATTCCTGTT-3′), *GAPDH* (forward: 5′-AAGAGCACAAGAGGAAGAGAGAGAC-3′, reverse: 5′-GTCTACATGGCAACTGTGAGGAG-3′). Cycling parameters included initial denaturation at 95 °C for 2 min, followed by 45 cycles of 95 °C for 15 s and 60 °C for 60 s. GAPDH was used as an internal control, and relative gene expression was calculated via the 2^−ΔΔCt^ method with technical triplicates [7].

### 2.4 Western blot analysis

For Western blot analysis, total protein was extracted using RIPA lysis buffer with protease inhibitors. Protein concentrations were measured by BCA assay. Equal amounts of protein were separated by SDS-PAGE and transferred to PVDF membranes. Membranes were incubated overnight at 4 °C with primary antibodies against FST (1:500, Proteintech, 60060-1-IG), TGF-β1 (1:500, Servicebio, GB115750), BMP4 (1:500, Proteintech, 12492-1-AP), Smad3 (1:500, Servicebio, GB 150085), and GAPDH (1:5000, Servicebio, GB 15004). HRP-conjugated secondary antibodies (1:5000) were applied for 1 h. Protein bands were visualized using ECL substrate, and band intensity was quantified with ImageJ software.

### 2.5 Immunohistochemistry staining

Immunohistochemistry (IHC) staining was performed on paraffin-embedded skin sections following deparaffinization, rehydration, and citrate-based antigen retrieval. Sections were incubated with the same primary antibody against FST utilized in Western blot assays, followed by incubation with biotinylated secondary antibodies and DAB chromogenic development. Images of target dermal regions were captured at 400× magnification, with tissue filling the field of view and consistent background illumination across all photographs. Image-Pro Plus 6.0 software was used for quantitative analysis. For each section, three fields of view were selected to measure the integrated optical density (IOD) and corresponding positive pixel area. The average optical density (AOD) was calculated as AOD = IOD/positive pixel area. AOD was selected as the primary quantitative indicator for subsequent statistical analysis, while raw IOD values were retained for reference.

### 2.6 Statistical analysis

Statistical analyses were performed using GraphPad Prism 10. Intergroup comparisons were carried out via unpaired Welch's *t*-test. FDR correction (Benjamini–Hochberg) was used for multiple gene comparisons to estimate the false discovery rate. Differentially expressed genes were identified based on raw P < 0.05. Data are expressed as mean ± SD, with individual raw data points overlaid in the figures. Statistical significance was defined as raw *P* < 0.05.

## 3 Results

### 3.1 Differential gene expression in SG vs. NSG

The PCR Array covered a total of 96 genes within the TGF-β/BMP signaling pathway, among which 30 genes were differentially expressed (17 upregulated and 13 downregulated) between SG and NSG postpartum subjects. *FST* exhibited the largest expression reduction, with expression approximately 225.59-fold lower in SG skin compared with NSG skin (P < 0.05) (Fig. 1A).

**Fig. 1:**
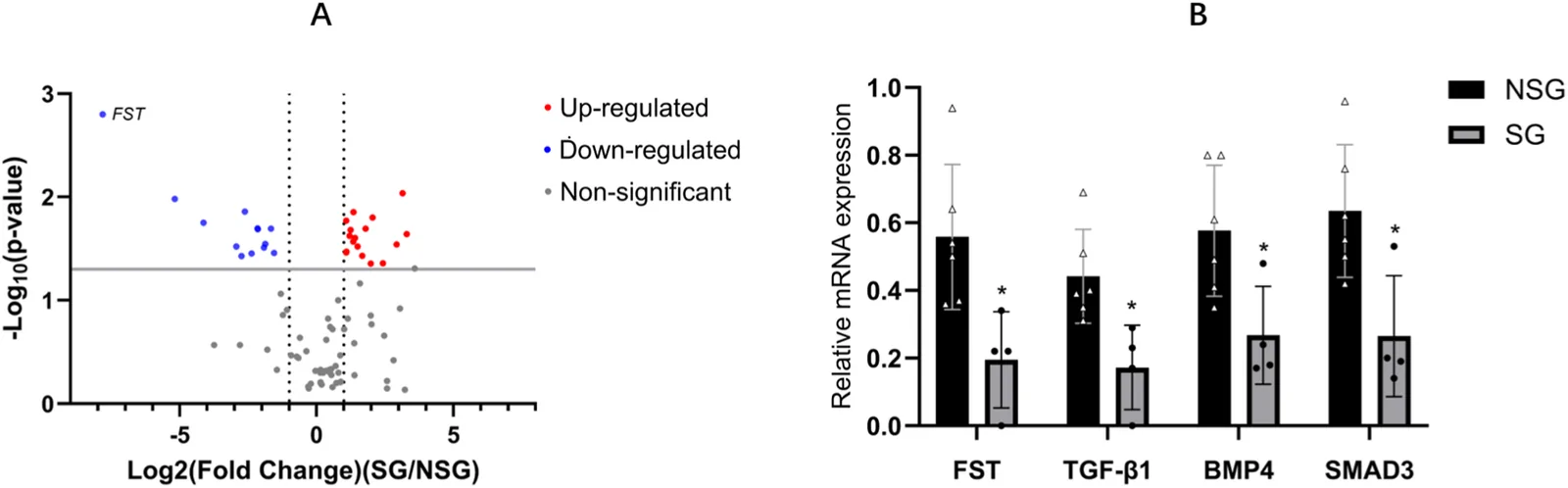
(A) Volcano plot of PCR Array data comparing gene expression between SG and NSG skin samples. The x-axis shows log_2_(fold change) (SG/NSG); negative values indicate reduced expression in SG. The y-axis represents –log_10_(raw p-value). Each dot represents a single gene. The most markedly dysregulated gene is annotated. Red dots: significantly upregulated genes in SG; blue dots: significantly downregulated genes in SG; grey dots: genes with no significant expression differences. (B) Quantification of relative mRNA expression of *FST, TGF-β1, BMP4* and *Smad3* in SG and NSG groups. Bars represent mean ± SD. Open triangles: NSG (n = 6); solid circles: SG (n = 4). *P < 0.05.

### 3.2 qPCR validation of key pathway genes

qPCR was further applied to verify the transcriptional changes of core pathway genes identified by PCR Array screening. Consistent with array results, the mRNA levels of FST, TGF-β1, BMP4 and SMAD3 were markedly lower in SG skin samples relative to NSG tissues (Fig. 1B). Relative expression values of each gene were normalized to GAPDH. Statistical analysis confirmed significant intergroup differences for all detected targets (*P* < 0.05).

### 3.3 Western blot analysis of target proteins

Western blot analysis was conducted to evaluate the protein abundance of FST, TGF-β1, BMP4 and Smad3 in SG and NSG skin tissues. Representative immunoblot bands are presented in Fig. 2 A, and densitometric quantification is shown in Fig. 2B. Densitometric quantification demonstrated that protein levels of all four target molecules were lower in SG lesions compared with NSG controls. Band intensity was normalized to GAPDH for each sample. (*P* < 0.05).

**Fig. 2:**
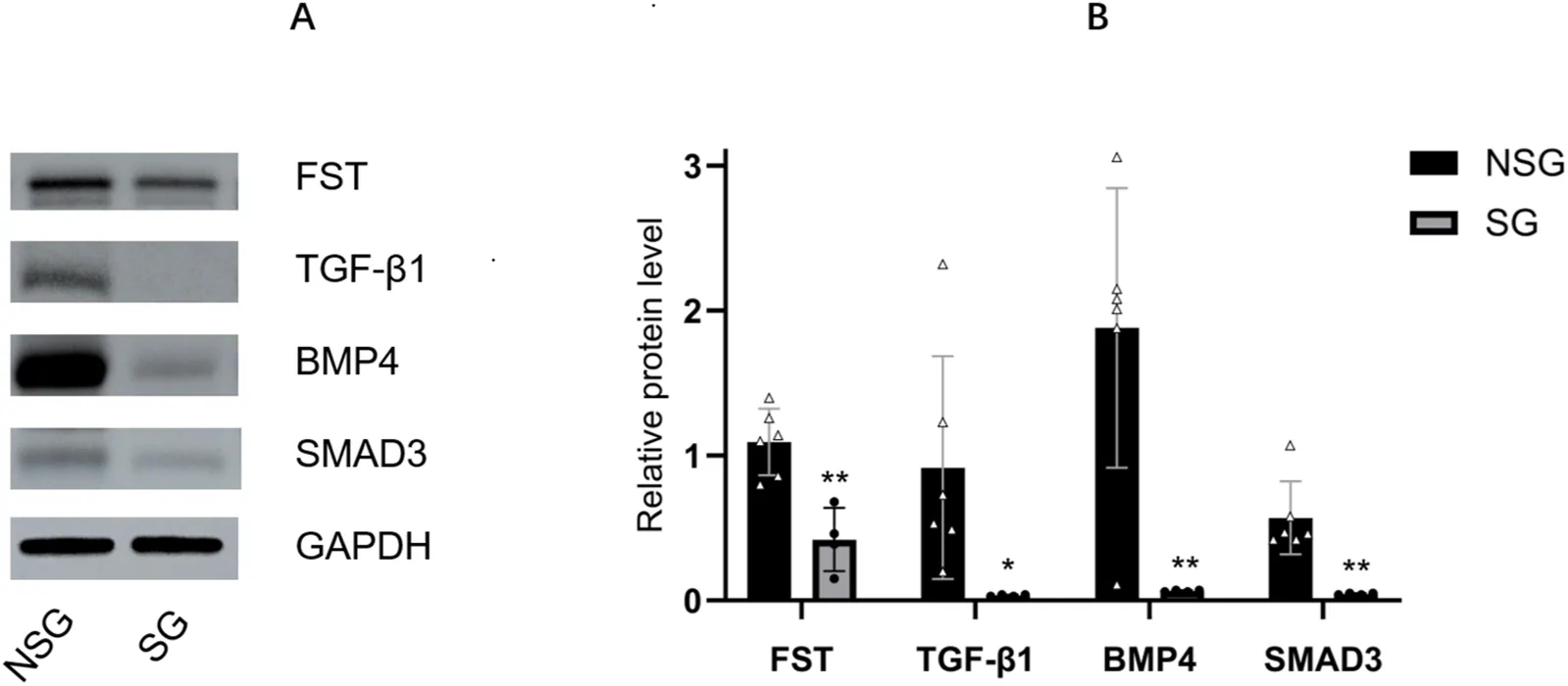
Western blot analysis of TGF-β/BMP pathway components (FST, TGF-β1, BMP4, Smad3) in NSG and SG skin. (A) Representative immunoblots, with GAPDH as internal control. (B) Densitometric quantification of relative protein levels. Bars show mean ± SD. Open triangles: NSG (n = 6); solid circles: SG (n = 4). *P < 0.05 and **P < 0.01.

### 3.4 IHC quantification of dermal FST expression

Immunohistochemistry (IHC) staining was performed to visualize the in situ distribution of FST within skin tissue sections. Positive FST immunoreactivity was predominantly observed in the dermal compartment. As illustrated in Fig. 3A and B, SG lesions displayed visibly weaker FST staining in the dermis when compared with NSG specimens. Quantitative analysis based on average optical density (AOD) was performed. This analysis confirmed lower FST expression in the SG group (P < 0.01). Raw IOD values were acquired during image processing for reference only.

**Fig. 3:**
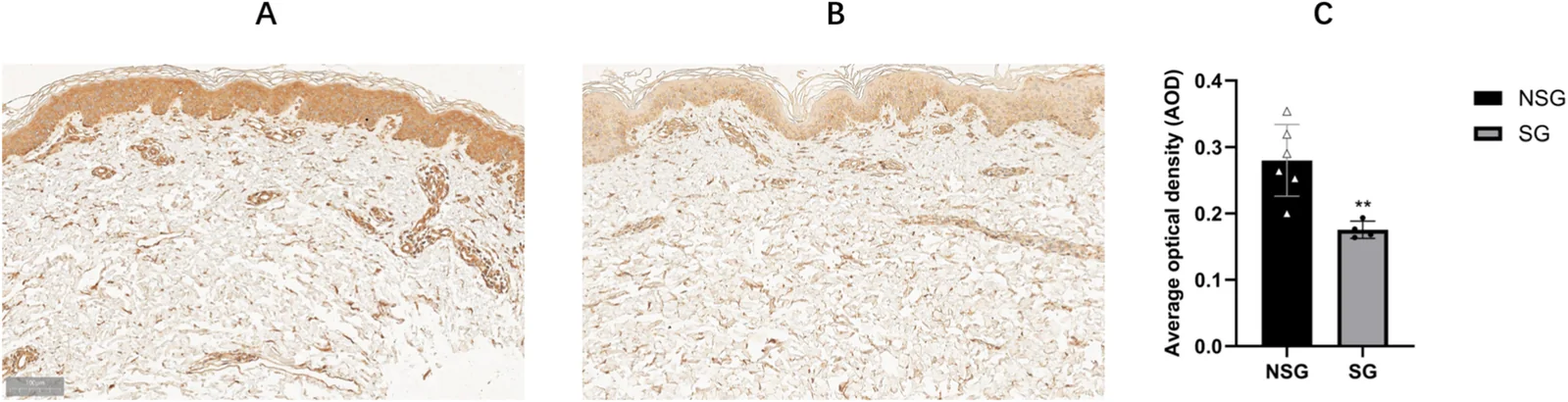
Immunohistochemical staining for FST in NSG and SG skin tissues. (A) Representative immunohistochemical images of FST in NSG skin; (B) Representative immunohistochemical images of FST in SG skin. Scale bar, 100 μm. Original magnification ×200. (C) Quantitative analysis of FST expression based on average optical density (AOD). Bars show mean ± SD. Open triangles: NSG (n = 6); solid circles: SG (n = 4). **P < 0.01.

## 4 Discussion

In the present study, we identified *FST* as the most markedly differentially expressed gene among TGF-β/BMP-related molecules in lesional SG skin. Both mRNA and protein levels of FST, TGF-β1, BMP4 and Smad3 were uniformly reduced in SG lesions, and immunohistochemistry (IHC) confirmed weakened dermal FST expression. As documented in prior literature, TGF-β1 initiates transcription of collagen and ECM genes to promote matrix synthesis and assembly, and also elevates elastin expression in dermal fibroblasts, placing it at the core of cutaneous collagen turnover [8], [9]. As an antagonist of multiple TGF-β superfamily ligands, low *FST* abundance may impair skin elasticity, making dermal tissue vulnerable to stretch-induced rupture [9]. We therefore speculate that, based on published mechanistic evidence, FST may bind activin A and participate in the modulation of TGF-β-related molecular cascades, which could affect downstream protein synthesis [10]. The coordinated downregulation of BMP4 and Smad3 alongside FST further suggests an association between reduced FST expression and altered abundance of TGF-β/BMP-related molecules. Additionally, keratinocyte-secreted FST is indispensable for epidermal repair [11], and reduced FST expression likely compromises epidermal regeneration and wound healing capacity. Taken together, existing literature indicates FST participates in dual regulatory roles in cutaneous scarring: it modulates scar formation and maintains collagen homeostasis via tuning of canonical TGF-β signal transduction.

Our results are consistent with existing data highlighting the key function of FST family proteins in skin repair and fibrosis. Sundaram et al. [12] reported that FSTL1 promotes keratinocyte proliferation, migration and re-epithelialization during wound healing. Ex vivo human skin studies further revealed that FSTL1 blockade attenuates TGF-β1-driven fibrotic changes [13]. Although these reports focused on FSTL1, our findings extend the known functional spectrum of this protein family and highlight the underexplored role of FST itself in striae gravidarum. It is well-established that keratinocyte-derived signals modulate fibroblast activity and shape the features of fibrotic skin scars [14], [15].

This work is the first to systematically map the expression pattern of FST-associated TGF-β/BMP-related molecules in human SG tissue, revealing a correlational molecular signature associated with SG pathogenesis. Still, several limitations warrant note. The small sample size restricts our conclusions to correlational associations, rather than definitive causal relationships. Furthermore, our analysis relies solely on human tissue expression data, lacking cellular or animal functional validation of pathway regulation. Future research will enlarge the sample cohort and perform cell-intervention experiments to explore whether FST can act as a viable therapeutic target for SG, providing novel directions for drug development and aesthetic management of striae gravidarum.

## CRediT authorship contribution statement

**Tian Tian:** Writing – original draft, Investigation, Funding acquisition, Conceptualization. **Huiyu Wang:** Validation, Methodology, Formal analysis. **Yuanhong Li:** Writing – review & editing, Supervision, Resources.

## Ethics declaration

Written informed consent to take part in the study and to publish the article has been obtained from all participants or their legal representatives. The privacy rights of participants have been observed.

This study included organ or tissue donors. This study includes human biological material and consent was obtained by donors, or their next of kin or legal representatives, for use in this study and for publication of the article. The samples used in this research were not sourced from executed prisoners or prisoners of conscience.

This study was performed in compliance with relevant laws, regulatory frameworks and guidelines where the research took place. This study was approved by the Institutional Medical Ethic and Human Research Committee of China Medical University. (Approval No. KLSH (2016) 2016-70-2)

## Funding

This work was supported in part by the 10.13039/501100004772Natural Science Foundation of Ningxia Province (Grant Number: 2023AAC03573).

## Declaration of competing interest

The authors declare that they have no known competing financial interests or personal relationships that could have appeared to influence the work reported in this paper.

## Data Availability

Data will be made available on request.
