# Supplementary material for: Low cutaneous follistatin expression is associated with striae gravidarum: A pilot study of the TGF-β/BMP signaling pathway
Source: Biochem Biophys Rep. 2026 Oct 13;48:102842. doi: 10.1016/j.bbrep.2026.102842 (PMC13685992; doi:10.1016/j.bbrep.2026.102842)
Supplement: Supplementary file 1 [file mmc1.zip › Legend.docx]

Figure S1: FST immunohistochemical micrographs of dermal tissue (epidermis excluded) used for quantitative analysis of dermal FST expression. Scale bar, 50 μm. Original magnification ×400. Images include 4 SG patients and 6 NSG controls, with three independent microscopic fields evaluated per patient. Image codes: SG#1-1, SG#1-2, SG#1-3 up to SG#4-1, SG#4-2, SG#4-3 (SG group). NSG#1-1, NSG#1-2, NSG#1-3 up to NSG#6-1, NSG#6-2, NSG#6-3 (NSG group). Each code denotes one microscopic field, and three codes correspond to the three separate fields obtained from one patient.
